# Arterial stiffness and blood pressure increase in pediatric kidney transplant recipients

**DOI:** 10.1007/s00467-022-05611-4

**Published:** 2022-09-12

**Authors:** Rizky Indrameikha Sugianto, Karen Ostendorf, Elena Bauer, Jeannine von der Born, Jun Oh, Markus J. Kemper, Rainer Buescher, Bernhard M. W. Schmidt, Nima Memaran, Anette Melk

**Affiliations:** 1 Department of Pediatric Kidney, Liver and Metabolic Diseases, Children’s Hospital, Hannover Medical School, Carl-Neuberg-Str. 1, 30625 Hannover, Germany; 2 University Children’s Hospital, University Medical Center-Hamburg-Eppendorf, Hamburg, Germany; 3 Department of Pediatrics and Adolescent Medicine, Asklepios Hospital Nord-Heidberg, Hamburg, Germany; 4 University Children’s Hospital, Essen University Hospital, Essen, Germany; 5 Department of Nephrology and Hypertension, Hannover Medical School, Hannover, Germany; 6 Children’s Hospital, Hannover Medical School, Carl-Neuberg-Str. 1, 30625 Hannover, Germany

**Keywords:** Arterial hypertension, Arteriosclerosis, Cardiovascular disease, Chronic kidney disease, Pulse wave velocity, Transplantation

## Abstract

**Background:**

Pulse wave velocity (PWV) is a measure of arterial stiffness. We investigated PWV and blood pressure (BP) to determine to what extent BP changes contribute to arterial stiffness, and secondly, to identify influencing factors on BP in children after kidney transplantation.

**Methods:**

Seventy children ≥ 2.5 years post-transplantation with at least two PWV measurements were included. Changes of systolic (Δ SBP) and diastolic BP (Δ DBP) were classified into “stable/decreasing,” “1–10 mmHg increase,” and “ > 10 mmHg increase.” Linear mixed modeling for PWV *z*-score (PWVz) adjusted either for Δ SBP or Δ DBP was performed. An extended dataset with monthly entries of BP, immunosuppression, and creatinine was obtained in 35 participants over a median of 74 months to perform linear mixed modeling for SBP and DBP.

**Results:**

PWVz increased with a rate of 0.11/year (95% *CI* 0.054 to 0.16). Compared to participants with stable BP, those with 1–10-mmHg SBP and DBP increase showed a higher PWVz of 0.59 (95% *CI* 0.046 to 1.13) and 0.86 (95% *CI* 0.43 to 1.30), respectively. A > 10-mmHg BP increase was associated with an even higher PWVz (SBP *β* = 0.78, 95% *CI* 0.22 to 1.34; DBP *β* = 1.37, 95% *CI* 0.80 to 1.94). Female sex and participants with lower eGFR showed higher PWVz.

In the extended analysis, DBP was positively associated with cyclosporin A and everolimus trough levels.

**Conclusions:**

A higher increase of PWV is seen in patients with greater BP increase, with higher cyclosporin A and everolimus trough levels associated with higher BP. This emphasizes the role of BP as a modifiable risk factor for the improvement of cardiovascular outcome after transplantation.

**Graphical abstract:**

**Figure:**
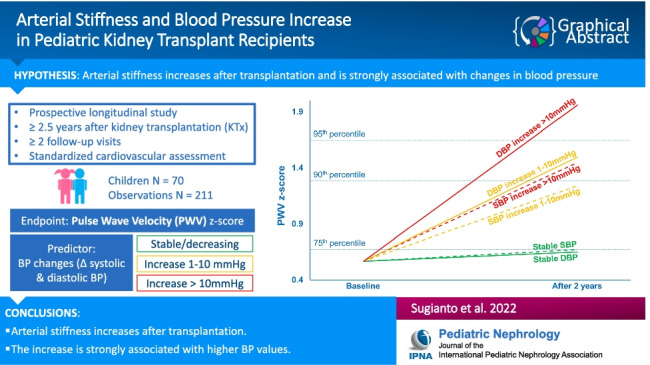
A higher resolution version of the Graphical abstract is available as Supplementary information

**Supplementary Information:**

The online version contains supplementary material available at 10.1007/s00467-022-05611-4.

## Introduction

Cardiovascular mortality in children with kidney failure is very high. About 30% of deaths in children on dialysis and 25% in children undergoing kidney transplantation (KTx) are due to cardiovascular events [1]. Data from the Australian and New Zealand Dialysis and Transplant Registry show an even higher cardiovascular mortality rate after KTx with 40% [2]. Importantly, mortality due to cardiovascular causes is higher than mortality due to non-functioning grafts [3].

The scarcity of cardiovascular mortality as well as major cardiovascular events in pediatric patients with chronic kidney disease (CKD) makes these endpoints unsuitable for studies in children. Arteriosclerosis, a marker for subclinical cardiovascular damage, is the process where elastic fibers are replaced by stiffer collagenous fibers after they break down due to daily wear and tear, which is eventually followed by the calcification of the media layer. This process is a major element in the stiffening of large arteries, which can be assessed by pulse wave velocity (PWV). PWV is highly predictive of major cardiovascular events and cardiovascular mortality in adults [4]. Although its predictive value has not been demonstrated in children, PWV measurement in the setting of clinical studies is recommended by the American Heart Association [5]. The procedure can be performed non-invasively and is highly reproducible in children [6, 7]. Furthermore, PWV is shown to be elevated in pediatric patients with CKD as well as after KTx, when compared to healthy peers [8–12].

A strong dependency between arterial stiffness and blood pressure (BP) is established for healthy children [13, 14] and children with CKD of all stages [10, 15–17], as well as in children after KTx [11, 18, 19]. In adults, not only the given BP levels but also the dynamic changes of BP over time have been shown to affect cardiovascular events [20]. In adult KTx recipients, a temporary BP increase is associated not only with worse patient survival, but also with inferior graft survival [21]. In fact, a 10-mmHg BP increase in patients with a systolic BP of > 140 mmHg is associated with a 12% higher risk of graft failure [22], and a 20-mmHg increase from a baseline systolic BP is associated with a 32% higher risk of subsequent cardiovascular disease [23]. In healthy male adults, the annual change in BP is positively associated with the progression of arterial stiffness [24], while a decrease in BP is associated with a lower PWV in patients with hemodialysis [25]. The association between BP dynamics and PWV has not been described in children yet.

We aimed to decipher the association between BP changes and progression of arterial stiffness. Our specific goal was to determine a clinically applicable BP range that may aggravate arterial stiffness in pediatric KTx recipients late after transplantation. By using an extended dataset with monthly entries over a period of up to 9 years, we aimed to further explore which factors are potentially associated with higher BP in a sub-cohort of our patients.

## Methods

### Subjects and study design

From January 2011 to August 2019, 124 pediatric KTx recipients from three German pediatric nephrology units (University Hospital Essen *n* = 41, University Medical Center Hamburg-Eppendorf *n* = 22, and Hannover Medical School *n* = 61) were enrolled in this prospective multicenter cohort study [18] after obtaining written informed consent from parents or caregivers. While per study protocol the only inclusion criteria were functioning kidney graft and age between 6 and 18 years, this analysis focused on patients with two or more follow-up visits ≥ 2.5 years after transplantation. The exclusion criteria were active systemic vasculitis, renal vascular anomalies, coexisting primary cardiovascular anomalies, and anomalies of the limbs preventing diagnostic procedures (which did not apply to any participant). The study was approved by each center’s ethics committee and fully adheres to the Declaration of Helsinki.

### Primary dataset

Aortic PWV was assessed every 2 years by two jointly trained investigators as previously reported [7] using the oscillometric Vicorder device (SMT Medical, Würzburg, Germany) in accordance with the recommendation of the Task Force III on clinical applications of arterial stiffness [26]. In short, three consecutive measurements with a length of at least 10 heart beats were performed in a supine position after 10 min of rest and then averaged. Blood and urine samples were obtained on the PWV measurement day and analyzed by a central laboratory (Synlab, Heidelberg, Germany), including whole blood count, creatinine, cystatin C, urea, and immunosuppressive trough levels. Creatinine was analyzed enzymatically, and immunosuppressive trough levels were analyzed using liquid chromatography and tandem mass spectrometry. Information on underlying disease, transplantation and dialysis history, and medication were obtained from the medical charts. Antihypertensive medication was classified into angiotensin-converting enzyme (ACE) inhibitors, angiotensin II receptor antagonists, β-blockers, calcium channel blockers, diuretics, vasodilators, α-blockers, and central acting agents. Measurement of office BP at each unit was performed on the same day by a pediatric nurse on the right arm in a seated position after 5 min of rest, using a validated oscillometric device (Hannover and Essen: Dinamap V100, GE Medical Systems, Chicago, IL, USA; Hamburg: Mindray VS-900, Mindray Bio-Medical Electronics, Shenzhen, China) with an appropriately sized cuff.

### Extended dataset

An extended dataset from the Hannover patients comprising monthly BP measurements, height, BMI, creatinine, and immunosuppressive trough levels was collected from medical records.

### Endpoints

As our primary endpoint, PWV measurements were transformed to standardized score (*z*-score) adjusted for sex and height [6]. Further endpoints in our extended dataset were systolic and diastolic BP.

### Covariates

We calculated age-, height-, and sex-standardized *z*-score for BP [27, 28], as well as age- and sex-standardized *z*-score for BMI. The estimated glomerular filtration rate (eGFR) was calculated using the Schwartz formula [29]. Immunosuppressive agents were classified as calcineurin inhibitors (CNI, including cyclosporin A or tacrolimus), mycophenolate mofetil (MMF), mammalian target of rapamycin inhibitors (m-TOR, including everolimus, and sirolimus), and steroids. Underlying primary kidney disease was classified as either congenital anomalies of the kidney and urinary tract (CAKUT) or primary kidney disease other than CAKUT (non-CAKUT).

The change of BP was calculated as the difference between the BP at each given visit and the BP at the first visit at ≥ 2.5 years after transplantation (baseline visit). We classified the change of BP into three categories: “stable/decrease,” “1–10 mmHg increase,” and “ > 10 mmHg increase.” Hypertensive BP was defined as systolic and/or diastolic BP ≥ 95th percentile (*z*-score ≥ 1.645) for patients with age < 16 years, or as a systolic/diastolic BP ≥ 140/90 mmHg for those ≥ 16 years old, or if the patient was on antihypertensive medication [28]. A non-hypertensive BP without antihypertensive medication was classified as “non-hypertension.” Further classification included “controlled hypertension” (non-hypertensive BP and treated), “uncontrolled hypertension” (hypertensive BP and treated), and “untreated hypertension” (hypertensive BP and untreated).

### Statistical analysis

Continuous variables are summarized by means (± standard deviation, SD) and categorical variables by frequencies and percentages. Differences between two or more independent groups were assessed using ANOVA or χ^2^ test for categorical variables. To investigate the effect of BP changes on PWVz, we performed a multivariable linear mixed effect modeling (mixed modeling) for PWV *z*-score (PWVz) at follow-up visits (after baseline visits), adjusted for sex [6, 12, 30], underlying disease, and baseline value of BP, as well as age [6], BMI [31], and eGFR [32] at the respective follow-up visit. Using the extended dataset, two mixed model analyses were performed, each for systolic and diastolic BP to investigate the associations with immunosuppressive trough levels (cyclosporin A, tacrolimus, everolimus), adjusted for sex [30, 33], and underlying disease, as well as age [30], BMI [34], and eGFR at the respective visits. Random intercept and slope to account for inter-individual variation and time since baseline visit as repeated effect to account for intra-individual variation were included. A two-tailed *p*-value of < 0.05 was considered statistically significant. Statistical analysis was performed using SAS 9.4 (SAS Institute, Cary, NC, USA).

## Results

### Patient characteristics

#### Primary dataset

Of 124 pediatric KTx recipients, 70 (*n* = 43 males) with at least two visits were included, resulting in 211 observations. At baseline, the age was 12.6 ± 3.2 years and elapsed time since transplantation was 6.1 ± 3.1 years. Participants were followed up for 4.0 ± 2.0 years with a maximum follow-up of 8 years. Fifty-one participants were transplanted after prior dialysis. The mean eGFR at the baseline visit was 56.6 ± 26.5 mL/min/1.73 m^2^. Detailed participant characteristics are given in Table 1. Supplemental Figure S1 provides the flow diagram of the study population in the primary dataset.

**Table 1:** Patient characteristics at transplantation and at study inclusion

At transplantation	*N*	%
All	70	100
Boys	43	61
Underlying renal diseases		
CAKUT	44	63
Non-CAKUT	26	37
Transplantation		
Preemptive transplantation	19	27
Transplantation with prior dialysis	51	73
Center		
Essen	18	26
Hamburg	13	19
Hannover	39	56
	*N*	Mean ± SD
Age at transplantation (years)	70	6.5 ± 4.2
At first visit ≥ 2.5 years after transplantation (baseline)		
Age (years)	70	12.6 ± 3.2
Time since transplantation (years)	70	6.1 ± 3.1
Height (cm)	70	147.7 ± 16.2
Height *z*-score	70	− 0.57 ± 0.97
BMI (kg/m^2^)	70	19.3 ± 4.0
BMI *z*-score	70	0.04 ± 1.12
Systolic BP (mmHg)	69	115 ± 11
Systolic BP *z*-score	69	0.86 ± 0.92
Diastolic BP (mmHg)	69	69 ± 10
Diastolic BP *z*-score	69	0.64 ± 0.85
Creatinine (mg/dL)	70	1.29 ± 0.61
Cystatin C (mg/L)	70	1.7 ± 0.6
Urea (mg/dL)	70	27.3 ± 14.8
eGFR (mL/min/1.73 m^2^)	70	56.6 ± 26.5
	*N*	%
Use of antihypertensive medication	60	86
Angiotensin-converting enzyme (ACE) inhibitors	40	57
Calcium channel blockers	38	54
β-blockers	36	51
Angiotensin II receptor antagonists	13	19
Diuretics	8	11
Vasodilators	8	11
α-blockers	3	4
Central acting agents	2	3
Use of immunosuppressive medication	70	100
Cyclosporin A	39	56
Tacrolimus	31	44
Mycophenolate mofetil	24	34
Mammalian target of rapamycin inhibitors	29	41
Steroids	28	40
Lipid lowering agent		
Statin	7	10

*BMI*, body mass index; *BP*, blood pressure; *CAKUT*, congenital anomalies of the kidney and urinary tracts; *eGFR*, estimated glomerular filtration rate; *SD*, standard deviation

**Fig. 1:**
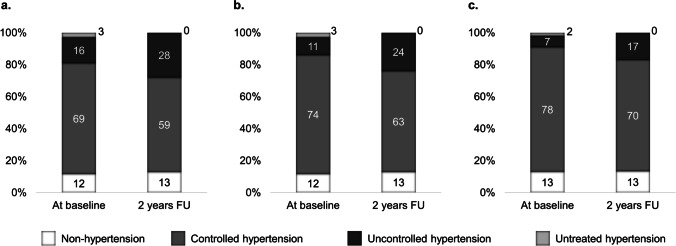
The proportion of hypertension (**a**), isolated systolic hypertension (**b**), and isolated diastolic hypertension (**c**) at baseline visit and 2-year follow-up. Abbreviation: *FU*, follow-up

Baseline systolic and diastolic BP *z*-scores were 0.86 ± 0.92 and 0.64 ± 0.85, respectively. Arterial hypertension was present in 88% of patients (69% controlled, 16% uncontrolled, and 3% untreated hypertension). The proportion of hypertension remained high over time, and the proportion of uncontrolled hypertension increased to 28% at the first follow-up visit (Fig. 1a) and could also be observed for further follow-up visits (data not shown). Figure 1b and c show the proportion of isolated systolic and isolated diastolic hypertension, respectively. Eighty-six percent of the participants received antihypertensive medication, which remained stable over time. The majority received a combination of three or more antihypertensive drugs (Supplemental Table S1), with ACE inhibitors, calcium channel blockers, and $\beta$-blockers as the most frequently used agents.

Of all participants, 97% had a combination of two (76%) or three (21%) immunosuppressive agents. The majority received a CNI-based regimen (97%), with CNI-mTOR, CNI-MMF, and CNI-steroids as the most commonly used combinations (Supplemental Table S2). At the baseline visit, the mean trough levels for cyclosporin A, tacrolimus, and everolimus were at 92.3 ± 29.3, 5.1 ± 1.8, and 3.9 ± 0.95 µg/L, respectively (Supplemental Table S2).

#### The course of PWVz

**Fig. 2:**
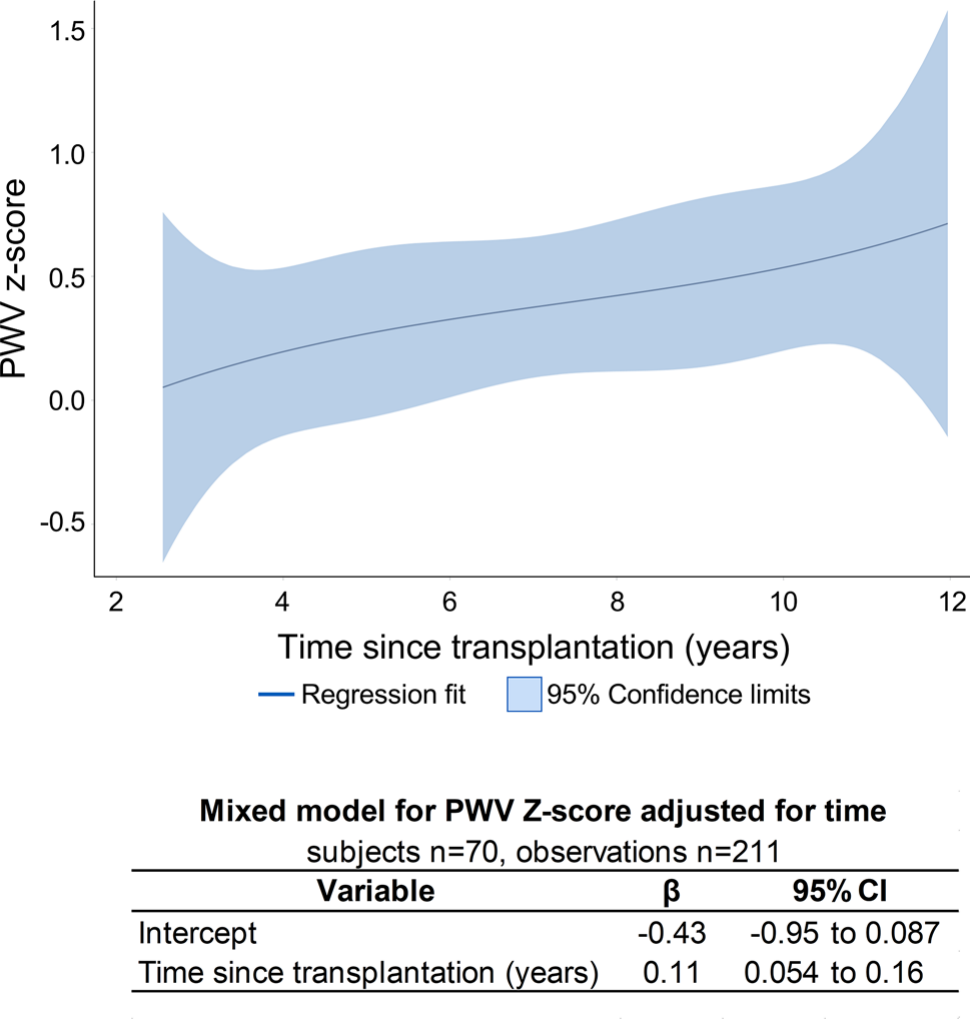
The longitudinal course of PWV *z*-score as visualized by regression spline (upper panel) and the mixed model for PWV *z*-score adjusted for time since transplantation (lower panel). Abbreviations: *β*, regression coefficient; *p*, *p*-value; *PWV*, pulse wave velocity

The mean of PWVz was 0.10 ± 1.31 at baseline and increased to 0.52 ± 1.26 after 2 years (Supplemental Table S3). The increase of PWVz was also demonstrated after adjustment for time since KTx with a rate of 0.11 per year (95% *CI* 0.054 to 0.16) (Fig. 2).

#### PWV and the changes of BP

Of 70 participants, 5 were excluded from the models due to missing BP at baseline and at following visits, and eGFR at following visits (Supplemental Fig. S1). In the model for PWVz and changes of systolic BP, when compared to participants with a stable/decreasing systolic BP, those with a systolic BP increase between 1 and 10 mmHg showed a higher PWVz increase of 0.59 (95% *CI* 0.046 to 1.13). A systolic BP increase of > 10 mmHg was associated with an even higher PWVz increase of 0.78 (95% *CI* 0.22 to 1.34). Significant associations between female sex, non-CAKUT underlying disease, and a lower eGFR with a higher PWVz were shown (Table 2).

**Table 2:** Mixed models for PWVz adjusted for changes in systolic blood pressure. Patients *n* = 65; observations *n* = 127

PWVz and changes in systolic BP		
Effect	*β*	95% C*I*
Intercept	− 1.85	− 4.81–1.11
Baseline systolic BP (mmHg)	0.019	− 0.01–0.05
Change of systolic BP (mmHg)		
1–10-mmHg increase	0.59	0.046–1.13
> 10-mmHg increase	0.78	0.22–1.34
Stable/decreasing	Ref	
Age (years)	0.050	− 0.037–0.14
Body mass index (kg/m^2^)	− 0.044	− 0.12–0.04
Girls (ref: boys)	0.55	0.003–1.10
Non-CAKUT (ref: CAKUT)	0.55	0.016–1.08
eGFR (mL/min/1.73 m^2^)	− 0.009	− 0.019–(− 0.0002)

*β*, regression coefficient; *BP*, blood pressure; *CAKUT*, congenital anomalies of the kidney and urinary tract; *CI*, confidence interval; *eGFR*, estimated glomerular filtration rate; *PWVz*, pulse wave velocity *z*-scores

The model for PWVz and changes in diastolic BP (Table 3) showed associations with even higher PWVz increases. Compared to children with stable/decreasing diastolic BP, children displaying an increase in diastolic BP between 1 and 10 mmHg showed a higher PWVz of 0.86 (95% *CI* 0.43 to 1.30). An increase in diastolic BP of > 10 mmHg was associated with a higher PWVz of 1.37 (95% *CI* 0.80 to 1.94). A higher PWVz was also associated with a higher baseline diastolic BP and female sex.

**Table 3:** Mixed models for PWVz adjusted for changes in diastolic blood pressure. Patients *n* = 65; observations *n* = 127

PWVz and changes in diastolic BP		
Effect	*β*	95% *CI*
Intercept	− 2.78	− 4.75–(− 0.82)
Baseline diastolic BP (mmHg)	0.047	0.024–0.07
Change of diastolic BP (mmHg)		
1–10 mmHg increase	0.86	0.43–1.30
> 10 mmHg increase	1.37	0.80–1.94
Stable/decreasing	Ref	
Age (years)	0.038	− 0.034–0.11
Body mass index (kg/m^2^)	− 0.051	− 0.11–0.014
Girls (ref: boys)	0.57	0.13–1.01
Non-CAKUT (ref: CAKUT)	0.41	− 0.020–0.83
eGFR (mL/min/1.73 m^2^)	− 0.008	− 0.017–0.0001

*β*, regression coefficient; *BP*, blood pressure; *CAKUT*, congenital anomalies of the kidney and urinary tract; *CI*, confidence interval; *eGFR*, estimated glomerular filtration rate; *PWVz*, pulse wave velocity *z*-scores

**Fig. 3:**
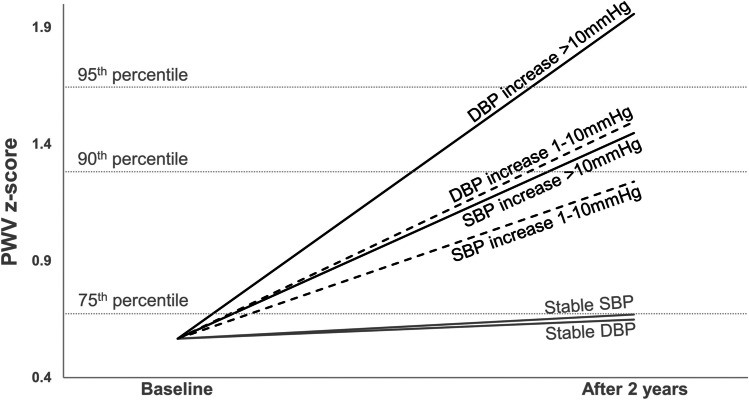
Illustration for the slopes of PWV *z*-score according to the defined categories of BP increases (stable/decreasing, 1–10 mmHg, or > 10 mmHg) in systolic or diastolic BP. The slopes are calculated according to the mixed models presented in Tables 2 and 3. Gray lines show the slopes for stable/decreasing systolic and diastolic BP. Black dotted lines show the slopes for systolic and diastolic BP increase of 1–10 mmHg. Black solid lines show the slopes for systolic and diastolic BP increase of > 10 mmHg. Abbreviations: *DBP*, diastolic blood pressure; *PWV*, pulse wave velocity; *SBP*, systolic blood pressure

In both models adjusted for changes in systolic and diastolic BP, age and BMI did not reveal a significant association with PWVz (Tables 2 and 3). We did not find associations between PWVz and immunosuppressive trough levels (Supplemental Table S4). Figure 3 illustrates the different PWVz slopes according to the defined categories of BP increases (stable/decreasing, 1–10 mmHg or > 10 mmHg) in systolic or diastolic BP.

#### Extended data analysis: blood pressure and immunosuppressive trough levels

We used an extended dataset to evaluate the importance of immunosuppression on BP. We retrieved data on systolic and diastolic BP, eGFR, and immunosuppressive trough levels from 35 participants (*n* = 20 males) comprising 2137 observations. The participants’ characteristics of the extended dataset are given in Supplemental Table S5. Tables 4 and 5 show the models for systolic and diastolic BP.

**Table 4:** Extended data analysis: mixed models for systolic BP

Systolic BP and immunosuppressive trough levels		
Patients *n* = 34*; observations *n* = 2015		
Effect	*Β*	95% *CI*
Intercept	72.95	61.11–84.80
Cyclosporin A trough level (µg/L)	0.025	− 0.002–0.051
Tacrolimus trough level (µg/L)	0.20	− 0.16–0.56
Everolimus trough level (µg/L)	0.13	− 0.24–0.49
Age (years)	1.33	0.81–1.86
Body mass index (kg/m^2^)	1.45	0.94–1.97
Girls (ref: boys)	1.22	− 12.10–14.53
Non-CAKUT underlying disease (ref: CAKUT)	− 2.96	− 16.38–10.46
eGFR (mL/min/1.73 m^2^)	− 0.02	− 0.070–0.029

*β*, regression coefficient; *BP*, blood pressure; *CAKUT*, congenital anomalies of the kidney and urinary tract; *CI*, confidence interval; *eGFR*, estimated glomerular filtration rate. *One patient (of 35) receiving sirolimus throughout the observation time was excluded from the models

**Table 5:** Extended data analysis: mixed models for diastolic BP

Diastolic BP and immunosuppressive trough levels		
Patients *n* = 34*; observations *n* = 2012		
Effect	*Β*	95% *CI*
Intercept	52.11	41.37–62.84
Cyclosporin A trough level (µg/L)	0.043	0.021–0.065
Tacrolimus trough level (µg/L)	0.21	− 0.082–0.51
Everolimus trough level (µg/L)	0.42	0.12–0.72
Age (years)	0.96	0.47–1.45
Body mass index (kg/m^2^)	0.33	− 0.10–0.77
Girls (ref: boys)	− 7.87	− 20.46–4.72
Non-CAKUT underlying disease (ref: CAKUT)	− 4.28	− 16.96–8.40
eGFR (mL/min/1.73 m^2^)	− 0.008	− 0.049–0.033

*β*, regression coefficient; *BP*, blood pressure; *CAKUT*, congenital anomalies of the kidney and urinary tract; *CI*, confidence interval; *eGFR*, estimated glomerular filtration rate. *One patient (of 35) receiving sirolimus throughout the observation time was excluded from the models

We did not find an association between immunosuppressive trough levels and systolic BP. Systolic BP increased with age. A higher BMI was associated with a higher systolic BP (Table 4).

In the model for diastolic BP, we found associations with higher trough levels of cyclosporin A (*β* = 0.043, 95% *CI* 0.021 to 0.065) and everolimus (*β* = 0.42, 95% *CI* 0.12 to 0.72). Diastolic BP increased with age (Table 5).

## Discussion

This prospective multicenter study on pediatric KTx recipients investigated PWV and its changes in dependence on BP changes at more than 2.5 years after transplantation. We demonstrated an increase in PWV late after transplantation that is associated with increasing BP values. We did not find associations between immunosuppressive trough levels and PWV. However, higher trough levels of cyclosporin A and everolimus were associated with higher diastolic BP values in our extended data analysis.

KTx resolves uremia, fluid overload, and other metabolic abnormalities, as reflected by the improvement of arterial stiffness observed in KTx recipients compared to patients on dialysis [11, 35, 36]. However, cardiovascular morbidity in this population remains high due to unresolved or newly developed risk factors such as hypertension, dyslipidemia, and obesity, exemplified by the high prevalence of post-transplant metabolic syndrome [37]. Compared to healthy children, arterial stiffness in pediatric KTx recipients with a functioning graft is higher [19] and further increases after transplantation [12, 38] indicating early development of arteriosclerosis. BP is a strong determinant for arterial stiffness in healthy children [13, 14] as well as those with CKD [10, 11, 18, 19]. In our longitudinal setup, we were able to identify the association between particular ranges of BP increases and the progression of arterial stiffness. An increase of 10 mmHg or more in systolic or diastolic BP was associated with a higher increase in arterial stiffness. Arterial hypertension is still highly prevalent and poorly controlled after KTx [39–42]. Whether strict BP control might reverse the pathological increase of arterial stiffness after KTx merits further research.

Our data did not show an association between different immunosuppressive drug trough levels and arterial stiffness. Randomized controlled trials in adults investigating the effect of different immunosuppressive regimens on arterial stiffness showed inconsistent results. A randomized controlled trial in 27 KTx recipients who were either switched from a CNI-based therapy to everolimus or remained on CNI therapy at 6 months after transplantation showed stable PWV in the everolimus group and a PWV increase in the CNI group at 15 months post-transplantation [43]. However, a larger prospective multicenter randomized controlled trial including 709 KTx recipients showed no differences in PWV between patients receiving standard therapy and those who switched to everolimus with a CNI-free regimen at 10–14 weeks after KTx [44]. We had previously demonstrated an association between the use of everolimus and higher PWV [18] and an association between higher tacrolimus or cyclosporin A trough levels with higher BP [42]. From our current findings, therefore, we speculate that the influence of everolimus and cyclosporin A on PWV is mediated through their effect on BP.

Our adjusted model suggested a possible association between female sex and a higher PWV *z*-score. This is consistent with our previous study demonstrating a greater vulnerability of female children with kidney failure and subsequent transplantation to develop arterial stiffness compared to males [12]. A study in adults demonstrated greater arterial stiffness in older females; however, this effect was attenuated with further adjustment for cardiovascular risk factors [45]. The possible association of lower eGFR with higher PWV is also in line with a previous study showing an association between faster eGFR decline and higher arterial stiffness [32]. The higher PWV in participants with underlying kidney diseases other than CAKUT might be explained by the association with a faster decline of kidney function shown in glomerular kidney disease [46] prior to transplantation. These exploratory observed associations between the covariates other than the primary exposure with the outcome variables are intriguing and warrant further investigations.

This study has some limitations. Longitudinal studies are subject to attrition bias; older participants in our cohort tended to have fewer follow-up visits due to their transition to adult care. However, PWVz did not differ between participants with 2 visits and those with > 2 visits. In addition, age was included in the mixed models. Our focus was to show how BP changes affect the PWV irrespective of the treatment strategies; we deliberately did not include variables indicating the use of antihypertensive medication. The lack of pre-KTx data in our dataset limits the exploration of potentially important factors, e.g., kidney functional decline during CKD progression. The data for the extended analysis was only available for Hannover patients. PWV and BP are highly predictive of cardiovascular outcome, but they are surrogate markers and not hard cardiovascular endpoints, such as myocardial infarction. We used single automated office BP measurements in our analysis. The nature of the observational study design does not allow to infer causality. Our study shows several strengths. The multicenter approach allowed us to include a high number of pediatric KTx recipients from three large German centers. All participants were followed longitudinally with standardized cardiovascular assessments. Our pediatric cohort is not confounded by age-related comorbidities as is usually the case in studies in adult populations, providing a clearer insight on cardiovascular changes.

## Conclusion

A higher burden of arterial stiffening with increasing BP of more than 10 mmHg as well as the association between higher cyclosporin A and everolimus trough levels with BP indicates the importance of modifiable risk factors for cardiovascular outcome after transplantation. It also emphasizes that there is room for improvement in BP control and highlights that the choice of immunosuppressive therapy can interfere with BP.

## Supplementary Information

Below is the link to the electronic supplementary material.

Graphical abstract (PPTX 179 kb)

(DOCX 32.7 KB)

## Data Availability

The datasets generated during and/or analyzed during the current study are available from the corresponding author on reasonable request.
