# Supplementary material for: Arterial stiffness and blood pressure increase in pediatric kidney transplant recipients
Source: Pediatr Nephrol. 2022 Sep 12;38(4):1319–27. doi: 10.1007/s00467-022-05611-4 (PMC9925540; doi:10.1007/s00467-022-05611-4)
Supplement: Supplementary file 1 — (PPTX 179 kb) [file 467_2022_5611_MOESM1_ESM.pptx]

## Slide 1
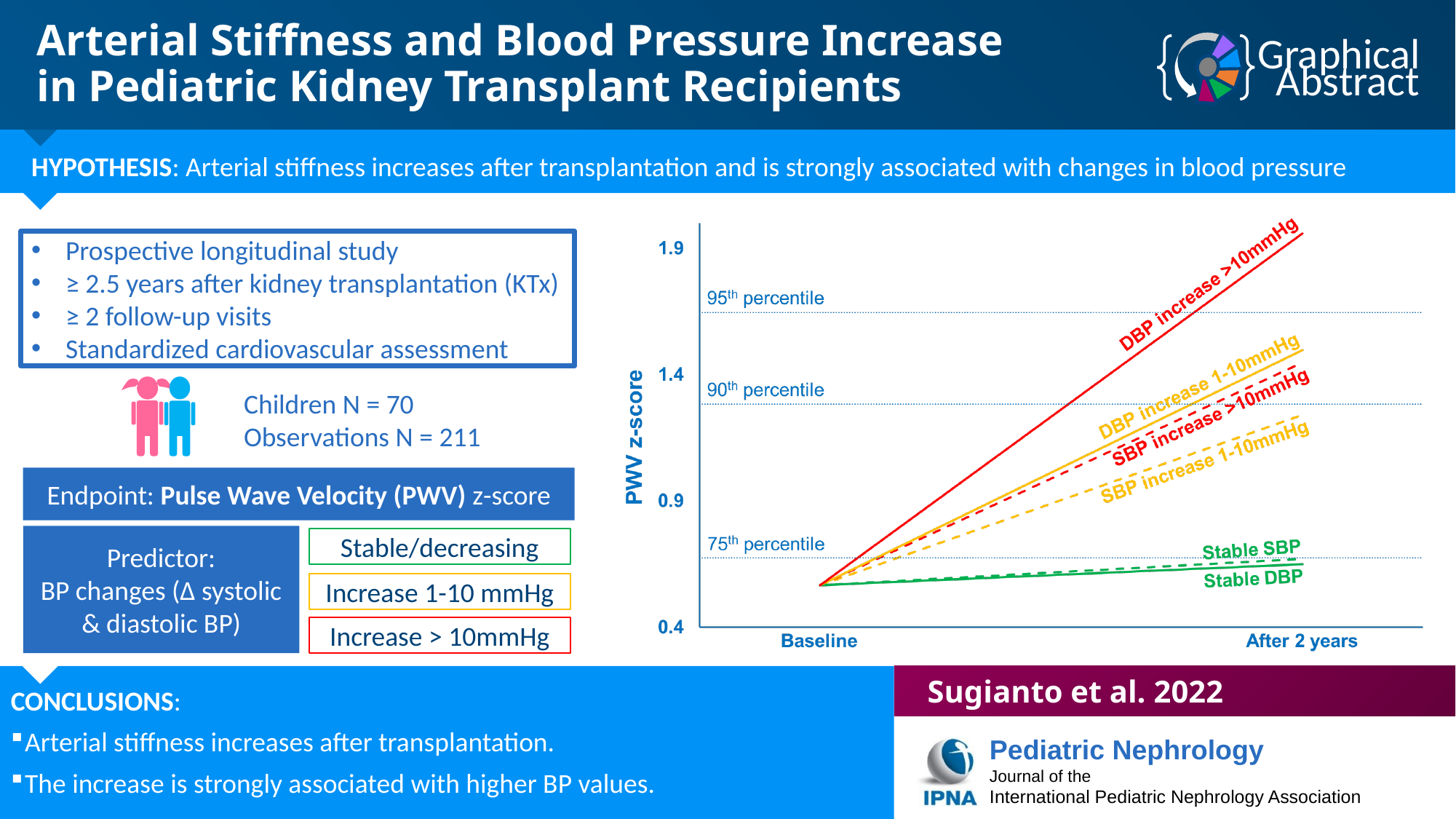

Arterial Stiffness and Blood Pressure Increase
in Pediatric Kidney Transplant Recipients
HYPOTHESIS: Arterial stiffness increases after transplantation and is strongly associated with changes in blood pressure
Prospective longitudinal study
≥ 2.5 years after kidney transplantation (KTx)
≥ 2 follow-up visits
Standardized cardiovascular assessment
Children N = 70
Observations N = 211
Endpoint: Pulse Wave Velocity (PWV) z-score
Predictor:
BP changes (∆ systolic & diastolic BP)
Stable/decreasing
Increase 1-10 mmHg
Increase > 10mmHg
Sugianto et al. 2022
CONCLUSIONS:
Arterial stiffness increases after transplantation.
The increase is strongly associated with higher BP values.
