# Supplementary material for: Arterial stiffness and blood pressure increase in pediatric kidney transplant recipients
Source: Pediatr Nephrol. 2022 Sep 12;38(4):1319–27. doi: 10.1007/s00467-022-05611-4 (PMC9925540; doi:10.1007/s00467-022-05611-4)
Supplement: Supplementary file 2 — (DOCX 32.7 KB) [file 467_2022_5611_MOESM2_ESM.docx]

| **Table 1** Patient characteristics at transplantation and at study inclusion | | |
| --- | --- | --- |
| **At transplantation** | **N** | **%** |
| **All** | 70 | 100 |
| **Boys** | 43 | 61 |
| **Underlying renal diseases** |  |  |
| CAKUT | 44 | 63 |
| Non-CAKUT | 26 | 37 |
| **Transplantation** |  |  |
| Preemptive transplantation | 19 | 27 |
| Transplantation with prior dialysis | 51 | 73 |
| **Center** |  |  |
| Essen | 18 | 26 |
| Hamburg | 13 | 19 |
| Hannover | 39 | 56 |
|  | **N** | **Mean ± SD** |
| Age at transplantation (years) | 70 | 6.5 ± 4.2 |
| **At first visit ≥2.5 years after transplantation (baseline)** |  |  |
| Age (years) | 70 | 12.6 ± 3.2 |
| Time since transplantation (years) | 70 | 6.1 ± 3.1 |
| Height (cm) | 70 | 147.7 ± 16.2 |
| Height z-score | 70 | -0.57 ± 0.97 |
| BMI (kg/m^2^) | 70 | 19.3 ± 4.0 |
| BMI z-score | 70 | 0.04 ± 1.12 |
| Systolic BP (mmHg) | 69 | 115 ± 11 |
| Systolic BP z-score | 69 | 0.86 ± 0.92 |
| Diastolic BP (mmHg) | 69 | 69 ± 10 |
| Diastolic BP z-Score | 69 | 0.64 ± 0.85 |
| Creatinine (mg/dL) | 70 | 1.29 ± 0.61 |
| Cystatin C (mg/L) | 70 | 1.7 ± 0.6 |
| Urea (mg/dL) | 70 | 27.3 ± 14.8 |
| eGFR (mL/min/1.73m²) | 70 | 56.6 ± 26.5 |
|  | **N** | **%** |
| **Use of antihypertensive medication** | **60** | **86** |
| Angiotensin-converting enzyme (ACE) inhibitors | 40 | 57 |
| Calcium channel blockers | 38 | 54 |
| β-blockers | 36 | 51 |
| Angiotensin II receptor antagonists | 13 | 19 |
| Diuretics | 8 | 11 |
| Vasodilators | 8 | 11 |
| α-blockers | 3 | 4 |
| Central acting agents | 2 | 3 |
| **Use of immunosuppressive medication** | **70** | **100** |
| Cyclosporin A | 39 | 56 |
| Tacrolimus | 31 | 44 |
| Mycophenolate mofetil | 24 | 34 |
| Mammalian target of rapamycin inhibitors | 29 | 41 |
| Steroids | 28 | 40 |
| **Lipid lowering agent** |  |  |
| Statin | 7 | 10 |
|  | | |
| Abbreviations: BMI, body mass index; BP, blood pressure; CAKUT, congenital anomalies of the kidney and urinary tracts; eGFR, estimated glomerular filtration rate; SD, standard deviation | | |

| **Table 2** Mixed models for PWVz adjusted for changes in systolic (a) and diastolic blood pressure (b). Patients n = 65; observations n = 127 | | | | | | |
| --- | --- | --- | --- | --- | --- | --- |
| 1. **PWVz and changes in systolic BP** | | | | | | |
| **Effect** | **β** | **95% CI** | | | | |
| Intercept | -1.85 | ­­-4.81 | | | – 1.11 | |
| Baseline systolic BP (mmHg) | 0.019 | -0.01 | | | – 0.05 | |
| Change of systolic BP (mmHg) |  |  | | |  | |
| 1-10 mmHg increase | 0.59 | 0.046 | | | – 1.13 | |
| >10 mmHg increase | 0.78 | 0.22 | | | – 1.34 | |
| Stable/decreasing | Ref. |  | | |  | |
| Age (years) | 0.050 | -0.037 | | | – 0.14 | |
| Body mass index (kg/m^2^) | -0.044 | -0.12 | | | – 0.04 | |
| Girls (ref: boys) | 0.55 | 0.003 | | | – 1.10 | |
| Non-CAKUT (ref: CAKUT) | 0.55 | 0.016 | | | – 1.08 | |
| eGFR (mL/min/1.73m²) | -0.009 | -0.019 | | | – (-0.0002) | |
|  |  |  |  |  | |  |
| 1. **PWVz and changes in diastolic BP** | | | | | | |
| **Effect** | **β** | **95% CI** | | | | |
| Intercept | -2.78 | -4.75 | | | – (-0.82) | |
| Baseline diastolic BP (mmHg) | 0.047 | 0.024 | | | – 0.07 | |
| Change of diastolic BP (mmHg) |  |  | | |  | |
| 1-10 mmHg increase | 0.86 | 0.43 | | | – 1.30 | |
| >10 mmHg increase | 1.37 | 0.80 | | | – 1.94 | |
| Stable/decreasing | Ref. |  | | |  | |
| Age (years) | 0.038 | -0.034 | | | – 0.11 | |
| Body mass index (kg/m^2^) | -0.051 | -0.11 | | | – 0.014 | |
| Girls (ref: boys) | 0.57 | 0.13 | | | – 1.01 | |
| Non-CAKUT (ref: CAKUT) | 0.41 | -0.020 | | | – 0.83 | |
| eGFR (mL/min/1.73m²) | -0.008 | -0.017 | | | – 0.0001 | |
| Abbreviations: β, regression coefficient; BP, blood pressure; CAKUT, congenital anomalies of the kidney and urinary tracts; CI, confidence interval; eGFR, estimated glomerular filtration rate; PWVz, pulse wave velocity z-scores | | | | | | |

| **Table 3** Extended data analysis: mixed models for systolic (a) and diastolic BP (b) | | | | |  |
| --- | --- | --- | --- | --- | --- |
| 1. **Systolic BP and immunosuppressive trough levels** | | | | |  |
| **Patients n = 34*; Observations n = 2,015** | | | | |  |
| **Effect** | | **Β** | **95% CI** | |  |
| Intercept | | 72.95 | 61.11 | – 84.80 | |
| Cyclosporin A trough level (µg/L) | | 0.025 | -0.002 | – 0.051 | |
| Tacrolimus trough level (µg/L) | | 0.20 | -0.16 | – 0.56 | |
| Everolimus trough level (µg/L) | | 0.13 | -0.24 | – 0.49 | |
| Age (years) | | 1.33 | 0.81 | – 1.86 | |
| Body mass index (kg/m^2^) | | 1.45 | 0.94 | – 1.97 | |
| Girls (ref: boys) | | 1.22 | -12.10 | – 14.53 | |
| Non-CAKUT underlying disease (ref: CAKUT) | | -2.96 | -16.38 | – 10.46 | |
| eGFR (mL/min/1.73m²) | | -0.02 | -0.070 | – 0.029 | |
| 1. **Diastolic BP and Immunosuppressive Trough Levels** | | | | |  |
|  | **Patients n = 34*; Observations n = 2,012** | | | |  |
| **Effect** | | **Β** | **95% CI** | |  |
| Intercept | | 52.11 | 41.37 | – 62.84 | |
| Cyclosporin A trough level (µg/L) | | 0.043 | 0.021 | – 0.065 | |
| Tacrolimus trough level (µg/L) | | 0.21 | -0.082 | – 0.51 | |
| Everolimus trough level (µg/L) | | 0.42 | 0.12 | – 0.72 | |
| Age (years) | | 0.96 | 0.47 | – 1.45 | |
| Body mass index (kg/m^2^) | | 0.33 | -0.10 | – 0.77 | |
| Girls (ref: boys) | | -7.87 | -20.46 | – 4.72 | |
| Non-CAKUT underlying disease (ref: CAKUT) | | -4.28 | -16.96 | – 8.40 | |
| eGFR (mL/min/1.73m²) | | -0.008 | -0.049 | – 0.033 | |
| Abbreviations: β, regression coefficient; BP, blood pressure; CAKUT, congenital anomalies of the kidney and urinary tracts; CI, confidence interval; eGFR, estimated glomerular filtration rate  * one patient (of 35) receiving sirolimus throughout the observation time was excluded from the models. | | | | |  |
